# Comparing post‐operative human breast specimen radiograph and MRI in lesion margin and volume assessment

**DOI:** 10.1120/jacmp.v13i6.3802

**Published:** 2012-11-08

**Authors:** Hiroyuki Abe, Akiko Shimauchi, Xiaobing Fan, Jonathan N. River, Husain Sattar, Jeffrey Mueller, Gregory S. Karczmar, Gillian M. Newstead

**Affiliations:** ^1^ Department of Radiology The University of Chicago Chicago IL USA; ^2^ Department of Pathology The University of Chicago Chicago IL USA

**Keywords:** breast lesion, MRI, specimen, mammography, margin assessment

## Abstract

The purpose of this research is to evaluate the potential for identifying malignant breast lesions and their margins on large specimen MRI, in comparison to specimen radiography and clinical dynamic contrast enhanced MRI (DCE‐MRI). Breast specimens were imaged with an MR scanner immediately after surgery, with an IRB‐approved protocol and with the patients' informed consent. Specimen sizes were at least 5 cm in diameter and approximately 1 to 4 cm thick. Coronal and axial gradient echo MR images without fat suppression were acquired over the whole specimens using a 9.4T animal scanner. Findings on specimen MRI were compared with findings on specimen radiograph, and their volumes were compared with measurements obtained from clinical DCE‐MRI. The results showed that invasive ductal carcinoma (IDC) lesions were easily identified using MRI and the margins were clearly distinguishable from nearby tissue. However, ductal carcinoma *in situ* (DCIS) lesions were not clearly discernible and were diffused with poorly defined margins on MRI. Calcifications associated with DCIS were visualized in all specimens on specimen radiograph. There is a strong correlation between the maximum diameter of lesions as measured by radiograph and MRI (r=0.93), as well as the maximum diameter measured by pathology and radiograph/MRI (r < 0.75). The volumes of IDC measured on specimen MRI were slightly smaller than those measured on DCE‐MRI. Imaging of excised human breast lumpectomy specimens with high magnetic field MRI provides promising results for improvements in lesion identification and margin localization for IDC. However, there are technical challenges in visualization of DCIS lesions. Improvements in specimen imaging are important, as they will provide additional information to standard radiographic analysis.

PACS numbers: 87.61.Tg; 87.59.B

## I. INTRODUCTION

Breast conservation therapy is a surgical treatment to remove malignant tumors with a small amount of tissue around it, and conserve the remaining healthy part of the breast. This is a standard treatment for breast cancer that is confined to less than one‐quarter of the breast volume.^(1)^ A key factor for successful conservation therapy is complete tumor resection without residual tumor cells in the breast. To check whether complete resection is achieved, currently the margins of the lumpectomy specimen are generally evaluated pathologically.^2^, ^3^

The pathologic method used in assessing the surgical margins, however, is a time‐consuming procedure, usually taking at least a few days to complete. Another issue with this method is that significant errors may occur because of the nature of the method. The possible errors include: (i) specimens are normally cut into 0.3–0.5 cm thick slices which could miss maximum tumor size or contiguous areas of tumor;^(4)^ (ii) tissue shrinkage and expansion from formalin fixation could vary tumor area/volume measurement;^(5)^ (iii) ink may seep into cracks or crevices in the surface of the tissue, including those created by tissue handling intraoperatively;^(6)^ and (iv) all margin evaluation is subject to sampling error, as complete sampling of the margin of a 2 cm specimen would require over 3000 sections.^(7)^ Therefore, a faster, more complete, and more precise method for assessment of the surgical margin is highly desirable.

Magnetic resonance imaging (MRI) has been implemented in the clinical practice of breast imaging for its ability to detect and visualize tumors far more sensitively and accurately than other conventional imaging modalities, such as mammography and ultrasound.^8^, ^12^ At our institution, MR imaging studies are nearly imperative for those patients who are candidates for breast conservation therapy for precise evaluation of the extent of the cancer. Considering its ability to precisely delineate the tumor, three‐dimensional high‐resolution MR images of the surgical specimen would be useful in visualizing the extent of the tumor in the specimen. Holland et al.^(13)^ demonstrated that *in vitro* (tissue sample less than 3 cm) high‐resolution breast MRI acquired at 1.5T with a special gradient coil correlated well with microscopic histology. This specimen imaging with MRI might allow accurate measurement of the distance between tumor edge and the surgical margin.^13^, ^17^ It also might help pathologists to perform clinical procedures more efficiently and rapidly, and to identify malignant tissue that might have been missed by conventional analysis.

In this research, we performed large breast specimen (>5 cm in diameter) MRI acquired at 9.4T small animal scanner to explore a new idea. The lesions detected on MRI were compared qualitatively with specimen radiographs, and the lesion volumes measured from specimen were compared with those measured from clinical dynamic contrast enhanced MRI (DCE‐MRI).

## II. MATERIALS AND METHODS

### A. Specimen

**Figure 1:**
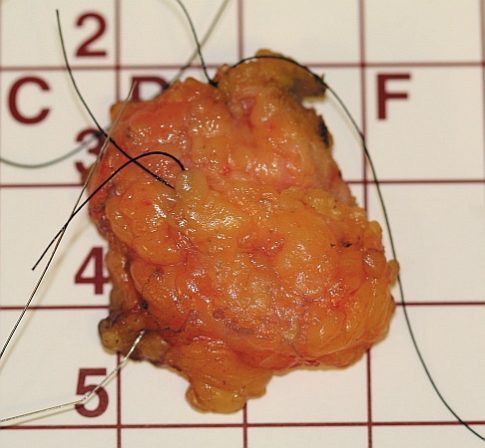
A fresh specimen placed on a grid board ready for imaging.

A total of 15 patients ((age=61±14), who were scheduled for lumpectomy, were recruited for this study under an Institutional Review Board (IRB)‐approved protocol and with patients' informed consent. Percutaneous biopsies and *in vivo* clinical DCE‐MRI were performed before surgery for all patients. Immediately following surgical excision, lumpectomy specimens were placed on a 12×20 cm special grid paper board (Fig. 1). Orthogonal mammograms of the specimens with magnification technique, without the paddle in place, were obtained using GE Senographe 2000D (GE Medical Systems, Milwaukee, WI) with a resolution of 57 micrometers, 25–26 kVp, and 50–71 mAs. For the magnification technique, we use a dedicated platform to increase object‐to‐image distance without a grid. The technique was adjusted for the thickness of the specimen and density of tissue. Then the specimens were kept in a container with ice and transported to the MRI research facility by a radiologist. The average specimen size was 7.8±1.8×6.1±1.1×2.2±0.9 cm in its length×width×height.

### B. Ex vivo MRI experiments

MRI experiments were conducted by using a 9.4T Bruker BioSpec 33 cm horizontal bore animal scanner (Bruker Daltonics Inc., Billerica, MA) with a 11.6 cm self‐shielded gradient coil insert (maximum strength 660 mT/m). The Bruker 72 mm birdcage volume coil was selected for all *ex vivo* imaging. Different MRI pulse sequences were tested on raw ground beef prior to the scans of specimens and the final optimized protocol was determined during the scan of the first specimen. Multislice coronal and axial gradient echo images without fat suppression (TR/TE=1000/7 ms, array size=256×256 for coronal and 256×128 for axial, flip angle=30°, slice thickness=1 mm, field of view=5 to 12 cm, resulting in‐plane resolution =∼0.47 to =∼0.20 mm, number of excitations=2) were acquired to cover the whole specimen. For larger specimens, two sets of axial images were acquired with 2 mm spacing and the center slice shifted by 1 mm in order to cover the whole specimen. Generally, the entire MRI experimental protocol did not exceed 45 minutes, including setup time, in order to provide the specimen to pathology in a reasonable time post‐surgery. Because the specimen was not removed from the grid board, both the specimen and board together were placed inside the imaging coil. As a result, there was some deformation in the MR images, as compared with the specimen radiograph.

Immediately after the MRI experiment, the specimen was sent to pathology for histopathology processing. Surgical specimens were fixed in a 10% formaldehyde solution and cut into serial 5 mm thick sections. Sections were embedded individually in paraffin wax to investigate the microscopic features. From every paraffin wax‐embedded section, one or several 5 μm thick slices were cut and stained with H & E. Shrinkage may have occurred during the process. For three cases, digital photographs of processed histopathology slices were also compared with specimen MRI in this study. Fixed gross specimens were sliced in the same plane as the specimen MR axial images.

### C. Data analysis

All *ex vivo* and clinical MRI data were analyzed using computer programs written in IDL (ITT Visual Information Solutions, Boulder, CO). The lesion type was verified by pathology. Two radiologists specialized in breast imaging performed a consensus reading of all specimen radiographs and specimen MR images, and compared them subjectively for visualization of tumors. The volume of the lesions in the specimen images was calculated by a summation of all slices with a lesion area manually traced on *ex vivo* MRI then multiplied by the slice thickness. To determine the volume of the lesions from *in vivo* clinical MRI, DCE‐MRI data between ~1 and ~3 min postcontrast injection was used. Location of the lesion was identified by interpreting images in the regular clinical manner, by using other relevant images such as mammography and ultrasound images, and a metallic clip placed after percutaneous biopsy, as a reference.

Clinical DCE‐MRI was performed using a 1.5T Signa scanner (GE Healthcare, Waukesha, WI) (n=6) or a 1.5T Intera Achieva scanner (Philips Healthcare, Andover, MA) (n=8). DCE‐MRI was not performed for one patient with diagnosis of radial scar. For the Signa scanner, the 3D fast gradient echo (FGRE) imaging parameters were as follows: TR/TE=4.6/2.2 ms, flip angle=10°, field of view (FOV)=34×34 cm, matrix=320×320, section thickness=2 mm, and temporal resolution=75 seconds. For the Intera Achieva scanner, the 3D fast field echo (FFE) imaging parameters were as follows: TR/TE=7.9/3.9 ms, flip angle=10°, FOV=48×48 cm, matrix=352×352, section thickness=2 mm, and temporal resolution=55 to 75 seconds.

## III. RESULTS

The lesion types identified by pathology and measurement information for all 15 specimens are given in Table 1. For comparing the maximum diameter of the lesions, 12 cancer cases in total were measured by pathology and radiograph; of those 12, 10 cases were also measured on specimen MRI, excluding two of the DCIS cases that were not visible on specimen MRI. To compare the volume of the lesions, measurements from clinical DCE‐MRI were compared with specimen MRI in nine invasive lesions, excluding all three of the DCIS cases, because lesion boundaries of DCIS were not clear on specimen MRI.

**Table 1:** Summary of all breast specimens imaged in this study, broken down by pathological type, with notes regarding the applicability to various measurements.

*Lesion Type*	*Number of Cases*	*Note*
Invasive ductal carcinoma (IDC)	7	Four cases had associated DCIS^a^
Ductal carcinoma *in situ* (DCIS)	3	Only one case was included as a measured maximum diameter on specimen MRI
Invasive lobular carcinoma (ILC)	1	None
Other invasive carcinoma	1	None
Lobular carcinoma *in situ* (LCIS)	1	Excluded from measurement
Lymph node	1	Excluded from measurement
Radial scar	1	Excluded from measurement

^a^ These DCIS cases were not included in size measurements.

**Figure 2:**
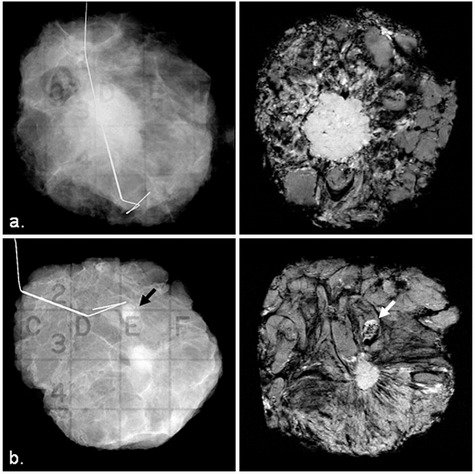
Comparison between specimen radiograph (left panel) and one selected slice from the MRI (right panel) for: (a) a 46‐year‐old patient with large IDC grade 3, and (b) a 44‐year‐old patient with small IDC grade 2 with a benign lymph node (arrows).

Figure 2 shows a comparison between the specimen radiograph (left panel) and one selected slice from the specimen MRI (right panel). All of the IDC lesions are easily identified on specimen MRI and the margins are clearly delineated from nearby tissue. The lesion margins are better visualized on specimen MRI than on specimen radiograph for six of the nine IDC lesions. For the remaining three IDC lesions, visibility of the lesion margins on specimen MRI is the same as on specimen radiograph.

**Figure 3:**
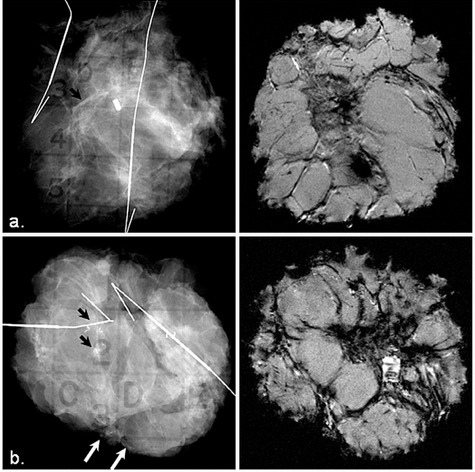
Comparison between specimen radiograph (left panel) and one selected slice from the MRI (right panel) for: (a) a 54‐year‐old patient with DCIS low nuclear grade, and (b) a 49‐year‐old patient with DCIS intermediate to high grade. The black arrows indicate clusters of calcifications targeted by one of the two wires, and white arrows indicate calcifications on the margin of the specimen.

**Figure 4:**
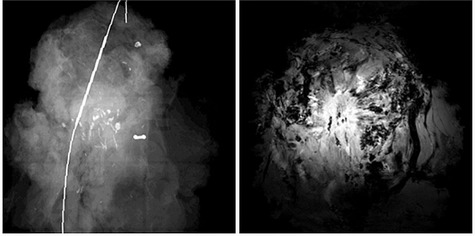
Comparison between specimen radiograph (left) and one selected slice from the MRI (right) for a 75‐year‐old patient with large IDC grade 2 and DCIS intermediate nuclear grade within IDC.

Figure 3 shows a comparison between specimen radiograph (left panel) and one selected slice from the specimen MRI (right panel). The calcifications representing DCIS can be seen clearly on the specimen radiographs in these two cases, but DCIS lesions cannot be clearly seen on the specimen MR images. The calcifications are visible on specimen MRI, when the DCIS component is surrounded by an IDC lesion (Fig. 4). Calcifications that were associated with DCIS were identified on specimen radiographs for all DCIS cases; however, DCIS lesions were poorly identified on specimen MRI. On specimen MRI, susceptibility artifacts from a marker

clip had a significant effect on image quality. In three of the nine invasive lesions, a part of the border of the invasive tumor was not well observed due to the clip artifacts.

**Figure 5:**
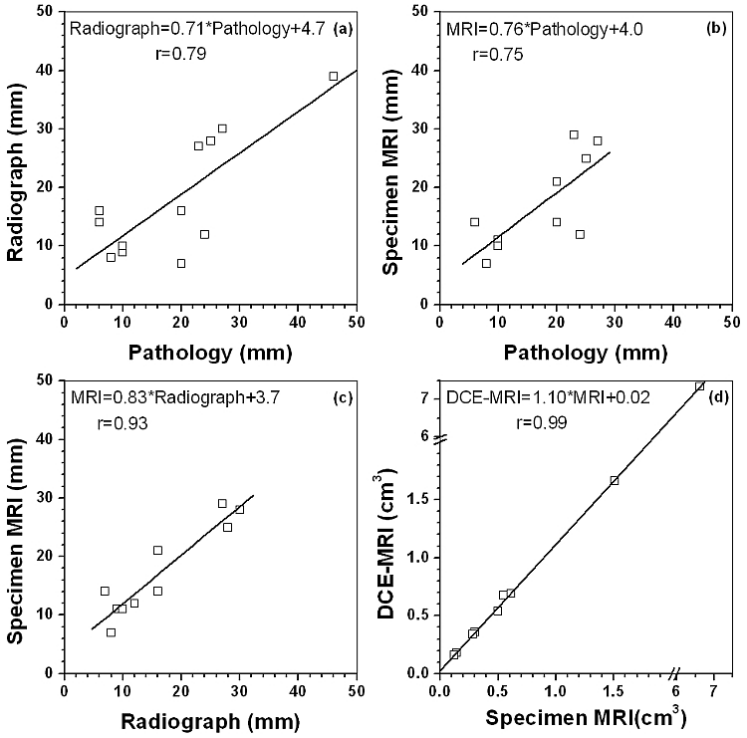
Comparison of the measured maximum lesion diameter between: (a) pathology and specimen radiograph, (b) pathology and specimen MRI, and (c) specimen radiograph and specimen MRI. Comparison of the measured lesion volume (d) between clinical DCE‐MRI and specimen MRI. The line in each graph is the linear fit to the data.

Measured maximum diameter from histology (all 12 cancer cases), specimen radiograph (all 12 cancer cases), and specimen MRI (10 cancer cases; two DCIS lesions were excluded) were compared and are shown in (Figs. 5a), (b), and (c). There were strong positive correlations in maximum diameter of lesions between histology and radiograph (r=0.79), and by histology and specimen MRI (r=0.75); but there was the strongest correlation between radiograph and specimen MRI (r=0.93). For lesion volume of the invasive lesions, there was very strong correlation (r=0.99) measured by clinical DCE‐MRI and specimen MRI ((Fig. 5d). However, lesion volume measured from specimen MRI was about 10%–30% smaller than from clinical DCE‐MRI, especially for small lesions (less than ~ $0.2\;{\text{cm}}^{3}$). This could simply be due to the difference in spatial resolution between specimen and clinical MRI. The volume of DCIS cannot be accurately assessed due to the failure of specimen MRI to detect clear lesion boundaries.

**Figure 6:**
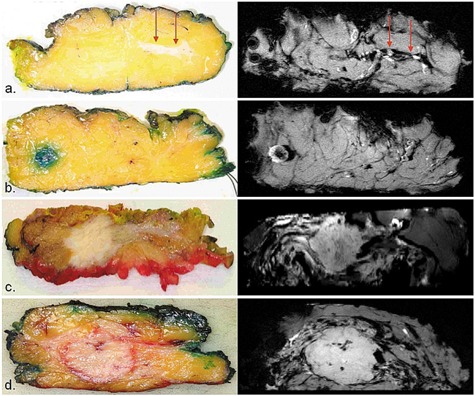
Examples of photographic pathology sections (left panel) with correlating axial slices of specimen MRI (right panel) of: (a) DCIS (red arrows), (b) lymph node, (c) IDC with DCIS, and (d) IDC.

Finally, Fig. 6 shows some examples of photographic pathology sections (left panel) with correlating axial slices of specimen MRI (right panel) of (a) DCIS, (b) lymph node, (c) IDC with DCIS, and (d) IDC. A DCIS component was visible in one of the three cases ((Fig. 6a). IDC lesions and lymph nodes were correlated well between pathology sections and MR images.

## IV. DISCUSSION

A total of 15 large breast specimens were imaged using a high strength magnetic field animal scanner and compared to specimen radiograph and clinical DCE‐MRI. The IDC lesions and lymph nodes could be easily detected on both specimen radiographs and specimen MRI. Nevertheless, specimen MRI provided better or equivalent lesion identification and margin localization for invasive carcinomas than for specimen radiograph. Our results demonstrated that the specimen radiograph could show all calcifications associated with DCIS; however, there are technical challenges in visualization of DCIS lesions for MRI. This is partially due to the fact that DCIS is composed of sparser tumor cells than IDC and, as a result, there is no well‐defined border in DCIS.

There is a strong correlation between maximum diameter of lesion as measured by radiograph and MRI. Therefore, both radiograph and MRI are useful in the prediction of tumor‐free margins. The volume of IDC detected on specimen MRI was fairly close to the volume measured on DCE‐MRI, when the difference in spatial resolution is taken into account. The reason is that there was about 10 times difference in area measurement alone due to average in‐plane resolution of 0.3 mm and 1.0 mm for specimen MRI and DCE‐MRI, respectively. In addition, morphological blooming due to contrast agent could blur the margin sharpness of lesion,^(18)^ and thereafter could affect the measurements of lesion volume in DCE‐MRI. Although MRI as one of the most sensitive imaging modalities is being widely used for detection and measurement of breast lesions, there was no “gold standard” in our lesion volume measurements.

The rationale of this study is to use specimen MRI for margin assessment, hopefully in the near future. Therefore, we conducted a pilot study to evaluate the visualization of tumor on selected slice from the specimen MRI in order to determine if it is comparable with — or perhaps better than — that of radiograph of the gross specimen. Although Holland et al.^(13)^ demonstrated correlation of high resolution specimen MRI with histopathology for small samples, more work is necessary, especially for technical development of specimen MRI for detecting DCIS in larger specimens. The protocols and techniques developed in this study could be further improved by developing better imaging sequences, using a dedicated MR coil, and using nonferromagnetic clips. We believe that DCIS could be better detected in MRI by using ultrashort TE pulse sequence to acquire super high‐resolution imaging; and the imaging time could be significantly reduced by using phased array coils. Precise pathologic correlation by slicing the pathologic specimen in the same plane as in the specimen MRI is imperative.

## V. CONCLUSIONS

In summary, our pilot study demonstrated that MRI of excised human breast lumpectomy specimens acquired with high magnetic field offers a promising way for lesion identification and margin localization for IDC. Further improvement is necessary in visualization of DCIS lesions in order to aid pathologists in routine assessment of specimens, thus contributing to better treatment of breast cancer patients. However, implementing specimen MRI is still far from realistic in the clinic because high field MRI scanners cost much more and are harder to operate compared to radiography. In addition, the imaging time for specimen MRI is much longer than specimen radiograph. Previous studies demonstrated that intra‐operative margin assessment in patients undergoing breast conserving surgery assisted in identifying positive/close margins and reduced re‐excision rates in patients.^19^, ^20^ Ideally, an MRI scanner should be used for intraoperative margin assessment (e.g., the MarginProbe^(21)^ (Dune Medical Devices, Gaithersburg, MD)), and it should be available for surgeons in real time to check the margins during breast‐conserving surgery.

## ACKNOWLEDGMENTS

This work was supported by a grant from the Lehman Foundation. We would like to thank Ms. Elizabeth Hipp for proofreading the manuscript.
